# A test of pre-exposure spacing and multiple context pre-exposure on the mechanisms of latent inhibition of dental fear: A study protocol

**DOI:** 10.1186/s40359-024-01580-5

**Published:** 2024-02-21

**Authors:** Andrew L. Geers, Laura D. Seligman, Keenan A. Pituch, Ben Colagiuri, Hilary A. Marusak, Christine A. Rabinak, Sena L. Al-Ado, Natalie Turner, Michael Nedley

**Affiliations:** 1 Department of Psychology, University of Toledo, 43606 Toledo, Ohio USA; 2 Department of Psychological Science, University of Texas Rio Grande Valley, Edinburg, Texas USA; 3 Edson College of Nursing and Health Innovation, Arizona State University, Phoenix, Arizona USA; 4 Department of Psychology, University of Sydney, Sydney, Australia; 5 Department of Psychiatry and Behavioral Neurosciences, School of Medicine, Wayne State University, Detroit, Michigan USA; 6 Department of Pharmacy Practice, Wayne State University, Detroit, Michigan USA; 7 Department of Dentistry, University of Toledo College of Medicine and Life Sciences, Toledo, Ohio USA

**Keywords:** Dental phobia, Latent inhibition, Fear learning, Pre-exposure, Pain sensitivity, Virtual reality, Eye tracking

## Abstract

**Background:**

Latent inhibition occurs when exposure to a stimulus prior its direct associative conditioning impairs learning. Results from naturalistic studies suggest that latent inhibition disrupts the learning of dental fear from aversive associative conditioning and thereby reduces the development of dental phobia. Although theory suggests latent inhibition occurs because pre-exposure changes the expected relevance and attention directed to the pre-exposed stimulus, evidence supporting these mechanisms in humans is limited. The aim of this study is to determine if two variables, *pre-exposure session spacing* and *multiple context pre-exposure*, potentiate the hypothesized mechanisms of expected relevance and attention and, in turn, increase latent inhibition of dental fear.

**Methods:**

In a virtual reality simulation, child and adult community members (ages 6 to 35) will take part in pre-exposure and conditioning trials, followed by short- and long-term tests of learning. A 100ms puff of 60 psi air to a maxillary anterior tooth will serve as the unconditioned stimulus. Pre-exposure session spacing (no spacing vs. sessions spaced) and multiple context pre-exposure (single context vs. multiple contexts) will be between-subject factors. Stimulus type (pre-exposed to-be conditioned stimulus, a non-pre-exposed conditioned stimulus, and an unpaired control stimulus) and trial will serve as within-subject factors. Baseline pain sensitivity will also be measured as a potential moderator.

**Discussion:**

It is hypothesized that spaced pre-exposure and pre-exposure in multiple contexts will increase the engagement of the mechanisms of expected relevance and attention and increase the latent inhibition of dental fear. It is expected that the findings will add to theory on fear learning and provide information to aid the design of future interventions that leverage latent inhibition to reduce dental phobia.

## Introduction

Dental phobia, a persistent and excessive fear of dental stimuli and procedures that results in avoidance or distress, represents a significant barrier to children and adult oral health [1]. Dental anxiety correlates positively with disadvantageous oral health behaviors and outcomes, including less frequent dental care visits [2, 3], lack of adherence to dentist advocated treatment [4], and increased likelihood of tooth decay, missing teeth, and periodontal diseases [5–7]. Dental fear and anxiety relate to poor oral health, which in turn, may increase the risk for heart disease, stroke, diabetes, and poor quality of life [8–10]. As marginalized individuals in the United States (including African American and Hispanic individuals) are particularly burdened by dental anxiety and problems of oral health, the study of dental anxiety has broad societal significance [11, 12].

Research suggests that direct associative conditioning is often responsible for the development of dental fear [13–15]. That is, an individual who undergoes an uncomfortable or painful dental procedure (unconditioned stimulus) may form an association between the dentist and/or dental-related stimuli (the conditioned stimulus) with fear (the conditioned response). In subsequent situations, such as future dental consultations, the dentist and/or dental related stimuli will then elicit the conditioned response.

Although experiencing painful or distressing dental events increases the likelihood of dental anxiety, this associative conditioning effect does not always occur. Research suggests that the experience of non-painful or non-traumatic dental events prior to negative dental experience(s) decreases the probability of enduring dental anxiety from associative conditioning [13, 14]. That is, pre-exposure to the dentist or dental related stimuli (e.g., office, chair, instruments) in the absence of a negative experience may reduce the likelihood of developing dental phobia. This impairment of associative learning from non-reinforced stimulus pre-exposure is a phenomenon labeled latent inhibition (LI). In LI, previous uneventful stimulus exposure reduces one’s ability to subsequently acquire or express a new association with that stimulus [16, 17].

It may be possible to strategically leverage the LI effect to help prevent the formation of dental phobia. To design effective interventions, however, it is critical to understand the mechanisms underlying LI of dental fear. This knowledge would allow for the creation of maximally efficient LI interventions—ones that generate clinically meaningful effects. As such, our team initiated a program of research to clarify the mechanisms underlying the LI of dental fear in humans. This work is grounded in the Hall and Rodríguez model of LI [18], which proposes expected relevance (i.e., prediction errors) and attention as mechanisms responsible for LI. According to this model, novel stimuli have high informational value and as such, they attract attention. When a novel stimulus is presented without an unconditioned stimulus (UCS), the heightened attention increases the likelihood the individual will learn a “stimulus → no event” association. When exposure to the stimulus continues without a UCS, the individual is said to receive further evidence for this stimulus → no event association. Over time, one learns that the pre-exposed stimulus is a signal with little informational value, and attention is therefore diverted elsewhere. When the stimulus is later paired with a UCS, the lack of attention hampers the associability potential of that pre-exposed stimulus, thereby resulting in LI.

Our team is testing the potential mediating role of expected relevance and attention in the LI of dental fear as proposed by the Hall and Rodríguez model of LI [18]. The present study will add to this line of investigation by experimentally testing if theoretically meaningful changes to pre-exposure modulate the mechanisms of expected relevance and attention, and in so doing change LI. Specifically, here we will investigate the variables of *multiple context pre-exposure* and *pre-exposure session spacing*. In terms of multiple context pre-exposure, presenting the pre-exposed stimulus with more than a single set of cues or settings should serve as a signal that the pre-exposed stimulus → no event association generalizes across contexts. This new learning should further decrease the associability of a stimulus, as this experience extends the initial learning to show the lack of stimulus relevance is not situation specific. Thus, context change should further reduce the expected relevance and attention to the pre-exposed stimulus, and therefore strengthen LI. Pre-exposure session spacing refers to temporally separating pre-exposure into discrete sessions. This spacing can be likened to a type of context change, a temporal change, and for the reasons just described should also decrease expected relevance and attention and thereby increase LI.

Prior studies are consistent with the perspective that pre-exposure spacing and pre-exposure occurring in multiple contexts changes LI. For example, animal studies and studies outside of the dental context, find that when pre-exposure occurs in multiple contexts, it can reduce fear at test and its return following extinction, as well as increasing the effectiveness of LI across contexts [19–21]. Animal studies also show that pre-exposure session spacing reduces fear learning and the context specificity of LI [19].

In addition to manipulating pre-exposure session spacing and context change, we will also test for moderation by individual differences in pain sensitivity. Prior research suggests that individual characteristics which rouse greater attention to a stimulus during pre-exposure should potentiate LI [22]. In the context of fear conditioning with an aversive UCS, pain sensitivity is such a variable [23].

## Research hypotheses

The proposed experiment will assess if pre-exposure session spacing and pre-exposure in multiple contexts potentiate changes in expected relevance and visual attention to the pre-exposed stimulus, and LI of dental fear. Pain sensitivity will also be measured to test if this variable moderates the influence of the proposed mediators on LI.

### Hypothesis 1

Spaced pre-exposure sessions, compared to no spacing, will (a) increase engagement of the proposed mechanisms (expected relevance and attention), and (b) enhance LI of conditioned fear acquisition, recall, and retention.

### Hypothesis 2

Multiple context pre-exposure, as compared to pre-exposure in a single context, will (a) lead to stronger engagement of the proposed mechanisms (expected relevance and attention), and (b) enhance LI of conditioned fear acquisition, recall, and retention.

### Hypothesis 3

Greater pain sensitivity will be associated with increased engagement of the proposed mechanisms of expected relevance and attention and increased LI.

## Methods

### Study participants

Participants will be 180 community volunteers between the age of 6 and 35, with this sample size based on an a priori power analysis for Hypotheses 1 and 2. The power analyses for Hypotheses 1a and 2a were focused on the simple pre-exposure-by-trial interaction for the conditioning and test phases. For the conditioning phase, use of G*power indicated a sample size of 19 is needed to detect a large effect, as suggested by our pilot study, (*f* = 1.3, α = 0.05, power = 0.80) whereas a sample size of 134 is needed for a given test phase to detect a small-to-moderate interaction (*f* = 0.20, α = 0.05, power = 0.80), where no prior effect size was available. For Hypotheses 1b and 2b, PASS software [24] indicated that a sample size of 106 is needed to achieve 0.80 power to detect an indirect effect equal to 0.15, where the standardized regression coefficient linking the mediator to the outcome is 0.3 and the correlation between the initiating variable and mediator is 0.5, with this correlation suggested by our pilot data.

Participants will be recruited in the regions surrounding the study sites in south Texas and northwest Ohio. It is expected that the study locations will provide the opportunity to recruit both Hispanic and African American participants, individuals that face increased burden from dental anxiety and poor oral health [11, 12]. Prior to entering the study, individuals will be screened using the inclusion/exclusion eligibility criteria displayed in Table 1. Participants will complete two or three in-person visits, depending on experimental condition, scheduled approximately 1 week apart. Participants will receive a $50 gift card for each visit. The Institutional Review Board of the primary institution approved the study.

**Table 1:** Exclusion and inclusion criteria

**Participant Exclusion Criteria**
Current injury (including fractures, open cuts, or sores) on their dominant hand
Currently have a cardiovascular disorder or a pacemaker
Currently have a seizure disorder or epilepsy
Fixed dental or orthodontic appliance that would interference with fabrication of a mouthpiece
Are colorblind
Currently on any anti-depressant or anti-anxiety medications
Currently have an inner ear infection
Living in the same household or an immediate family member of a participant enrolled in this or prior studies in this series
Unwilling to remove facial cosmetics or attend sessions without facial cosmetics on
Currently have glasses or use contact lenses with a vision correction of ≥ ± 6
History of sensitivity to extreme cold or frostbite
Bleeding disorder or take blood thinners
Vasospastic disorder such as Raynaud’s disease or Raynaud’s syndrome
Gastrointestinal or vestibular disorders that may elevate susceptibility to nausea and dizziness such as hyperemesis gravidarum, Meniere’s disease, or severe migraines
Behavioral, developmental, or sensory disorder that would increase discomfort or ability to complete study tasks
Medical condition that requires them to avoid mild stress
Diagnosis of Temporomandibular Disorder along with a history of exacerbation of symptoms resulting from routine dental procedures
Prior negative outcome with virtual reality simulation
Any medical condition that elevates risk of falls, nausea, dizziness, or causes vasovagal reactions
Any other oral or general health concerns
**Participant Inclusion Criteria**
Be between the ages of 6 and 35 years old
At least 2 of their maxillary anterior 6 teeth present
All of their maxillary anterior 6 teeth are free of hypersensitivity to pressure or pain
All of their maxillary anterior 6 teeth are stable
All of their maxillary anterior 6 teeth are free of cavities
Able to read, write, and converse in English (English or Spanish at UTRGV site)
Willing and able to provide a signed and dated informed consent/assent form to participate
Willing and available to comply with all study procedures and available for the study duration

### Design

The experiment employs a mixed research design, with two within-subject independent variables, two between-subjects independent variables, and one measured continuous variable. The within-subjects variables are stimulus type [stimulus type: pre-exposed to-be conditioned stimulus (CS^+^ _P_), non-pre-exposed to-be conditioned stimulus (CS^+^ _NP_), and an unpaired control stimulus (CS^−^)] and time [trial number]. The between-subjects variables are pre-exposure session spacing [no spacing vs. session spacing] and pre-exposure context [single context vs. multiple contexts]. Pain sensitivity will serve as a continuous moderator variable.

### Procedure

Individuals interested in the study will complete screening and screening consent on the phone or in a virtual meeting. Adults will screen for themselves, whereas a parent or legal authorized representative will screen for children and adolescents. Those qualifying after the screening of inclusion/exclusion factors will be scheduled for the first study session, with subsequent sessions occurring approximately one week apart from each other (min = 7 days). As described below, the number of sessions depends upon pre-exposure spacing condition.

At the start of session 1, participants will re-confirm eligibility, be provided a tablet device, and go through the informed consent (and assent, if appropriate) process. Parent or legal authorized guardian will provide permission for child and adolescent participants. Participants will then complete a pain sensitivity questionnaire and behavioral assessment of pain sensitivity, as described below. Researchers will then fabricate an individualized dental mouthpiece for the participant, place electrodermal activity electrodes on their hand, and deliver instructions for using the virtual reality set up. Using the randomization feature in REDCap (Research Electronic Data Capture; [25, 26]), participants will then be assigned to one of the four between-subject conditions. Randomization occurs during the first study visit, to maintain allocation concealment [27]. Participants will then begin the fear conditioning experimental task.

#### The experimental task

The experimental task occurs in a novel immersive virtual reality simulation depicting an unfamiliar alien landscape. The programmed was designed with the Vizard software [28] and participants will use the HTC Vive Pro headset and controllers to interact with the created environment [29]. The task will be described as a game in which the objective is to gather fuel canisters on an unfamiliar alien planet which will provide their spaceship the power it needs to get back to Earth. The simulation presents a large desert-like outdoor landscape with features such as unusual plants, rock arches, tall mountain cliffs, and alien artifacts. Critical for the experimental task, participants will encounter aliens on the planet surface. Participants will be informed that the alien inhabitants and their planet are unusual, and events may follow different rules than on Earth. They will be told that some of the interactions with aliens may be somewhat uncomfortable. These instructions are used to trigger schemas related to pain sensitivity, reflecting experiences at dental visits, while providing a rationale for the mouthpiece the participant will wear during the entire task. Further details of the experimental task and procedures are provided by Seligman et al. [23].

#### Task phases and manipulations

From the perspective of the participant, the virtual reality “game” will appear seamlessly in each session. However, the task consists of four distinct phases: pre-exposure, conditioning, and short- and long-term learning test phases. As described below (see Table 2), the phases occur on different days, depending on pre-exposure spacing condition.

**Table 2:** Trials in each session as a function of experimental condition

Experimental Condition	Session 1	Session 2	Session 3
No Session Spacing/ No Context Change	-12 pre-exposure trials in Context A -36 conditioning trials -12 fear recall trials	-12 fear retention trials	-------
Sessions Spaced/ No Context Change	- 6 pre-exposure trials in Context A or Context B	- 6 pre-exposure trials in Context A or Context B -36 conditioning trials -12 fear recall trials	-12 fear retention trials
No Session Spacing/ Context Change	- 6 pre-exposure trials in Context A; - 6 pre-exposure trials in Context B -36 conditioning trials -12 fear recall trials	-12 fear retention trials	-------
Sessions Spaced/ Context Change	- 6 pre-exposure trials in Context A or Context B	- 6 pre-exposure trials in Context B or Context B -36 conditioning trials -12 fear recall trials	-12 fear retention trials

*Note* Context A refers to an environment with a brighter tan color and lighting level, whereas Context B refers to an environment with a darker green color and lower lighting level.

The first trial phase, the *pre-exposure phase*, begins with a minimum of six seconds during which the participant can traverse freely in the virtual reality environment. Participants are tasked with locating fuel canisters that are scattered across the alien planet landscape. Following the initial pre-exposure trial segment, the CS^+^ _P_ is automatically displayed, accompanied by a fuel canister, at the first opportunity. The CS^+^ _P_ are alien stimuli that have small vertical motion and customized sounds. The CS^+^ _P_ and the fuel canister will appear at a fixed distance from the participant and will stay in that location for the final six seconds of the trial. Each trial therefore contains a six second segment without the CS^+^ _P_, followed by a six second segment with the CS^+^ _P_ and fuel canister. There will be twelve pre-exposure trials and during these trials the UCS is never presented.

The two between-subject independent variables are manipulated during the pre-exposure phase. The *pre-exposure context* independent variable has two levels: approximately half of the participants will complete the pre-exposure trials in a single context, whereas the other half will complete the pre-exposure trials in two contexts. Participants in the single context condition will complete all twelve pre-exposure trials in the same environment with a constant bright tan color and lighting level. Participants in the multiple contexts condition will complete six trials consecutively in the same bright tan color and lighting level as experienced by participants in the single context condition. The other six trials will occur consecutively with a darker green environment with a lower lighting level. Whether participants in the multiple contexts condition receive the six tan or green environment trials first will be counterbalanced. Next, the *pre-exposure session spacing* independent variable has two levels: no session spacing and session spacing. Participants in the no spacing condition will receive all twelve pre-exposure trials in their first experimental session. In contrast, participants in the spaced sessions condition will have six of the pre-exposure trials in their first experimental session, whereas the final six will occur at least 7 days later in a second session. The number of pre-exposure trials for all groups remains at twelve, to avoid confounding the number and spacing of trials. The allocation of trials across study sessions for each of the four between-subject conditions is displayed in Table 2.

Following the completion of the pre-exposure trials, participants will move to the *conditioning phase*, which will consist of 36 trials (12 CS^+^ _P_ trials, 12 CS^+^ _NP_, and 12 CS^-^ trials). The makeup of the conditioning trials will be the same as the pre-exposure trials, with the crucial difference that 75% of CS^+^ _P_ trials and 75% of CS^+^ _NP_ trials will co-occur with a dental startle UCS (explained below in the stimulus material section). The startle UCS will occur during the last 100ms of the trial and will co-terminate with the alien stimulus. The startle UCS will never be delivered on CS^-^ trials.

After the final conditioning trial, participants will move to the fear *recall phase*, which consists of a series of 12 test trials (4 CS^+^ _P_ trials, 4 CS^+^ _NP_, and 4 CS^-^ trials). The structure of the recall phase trials will be the same as in the prior phases. Importantly, the startle UCS will not accompany any of the alien stimuli in the fear recall phase.

Finally, all participants will return to the laboratory at least 7 days following the session that includes the conditioning phase and complete the fear *retention phase*. This phase will consist of 12 trials that have the same set up as those in the fear recall phase.

### Materials

#### Alien images

Three novel alien stimuli were created specifically for use in this virtual reality paradigm as the CS^+^ _P_, CS^+^ _NP_, and CS^-^. The stimuli were piloted with individuals between the ages of 6 and 35 to create stimulus images that are perceived as neutral and distinct from one another. Nevertheless, the three alien stimuli will be counterbalanced across participants for the CS^+^ _P_, CS^+^ _NP_, and CS^-^ to avoid confounding between alien image and stimulus type.

#### Dental startle UCS

This study will use a novel dental startle UCS previously described by Seligman et al. [23]. This stimulus is a 60 psi air puff that is delivered to a maxillary anterior tooth for 100ms through an individualized dental mouthpiece. The mouthpiece will be fabricated in visit 1 using 3 M™ STD Vinyl Polysiloxane Express Putty. The constructed mouthpiece will be used during the experimental task for all study sessions. The puff of air will be delivered to the mouthpiece with a 3/16 inch wide tubing that will receive pressurized air from a California Air Tools 8010 Steel Tank Air Compressor through an AIRSTIM device (San Diego Instruments, San Diego, California, USA). Participants will be told that the mouthpiece will allow them to experience the diverse sensations that humans can have on the alien planet.

### Measures

#### Mediating variables

We will assess *expected relevance* after the onset of the CS^+^ _P_, CS^+^ _NP_, or CS^-^ but before the onset of the startle UCS. The measure will appear in the virtual reality environment and the program will pause as participants rate the probability of a negative event occurring on a 10-point scale. Participants will use the handheld controllers to make their ratings.

To assess attention, we will track eye movements using software embedded in the Vive Pro headset. The amount of visual attention directed at the CS^+^ _P_, CS^+^ _NP_, and CS^-^ will be recorded [30, 31].

#### Subjective indicator of fear learning

The experiment will include indicators of subjective, physiological, and behavioral fear learning. In terms of subjective fear learning, participants will provide ratings of their relaxation/anxiety after the onset of the CS^+^ _P_, CS^+^ _NP_, or CS^−^ but prior to the onset of the dental startle UCS. The measure will be displayed in the virtual reality environment and participants will use the handheld controllers to make ratings [23].

#### Behavioral indicators of fear learning

We will include two indicators of behavioral fear learning. First, we will record the number of times the participant approached the CS^+^ _P_, CS^+^ _NP_, and CS^−^ and successfully obtained the “fuel cell” that appeared with alien. Second, we will measure the shortest distance between the participant and the CS^+^ _P_, CS^+^ _NP_, and CS^−^ on each trial [23].

#### Physiological indicator of fear learning

Finally, to provide a physiological index of fear learning, we will record skin conductance responses (SCRs) to the CS^+^ _P_, CS^+^ _NP_, and CS^−^. The Biopac systems MP160 with a wireless BioNomadix module transmitter (Biopac Systems, California, USA.) will be used for this assessment. SCRs will be captured with two Ag–AgCl electrodermal conductance electrodes with Isotonic gel placed on the middle phalanges of the non-dominant hand pinky and ring fingers. SCRs will be analyzed based on our prior work [32].

#### Moderator variables: pain sensitivity

We will assess *pain sensitivity* through self-report and a behavioral task. Self-reported pain sensitivity will be obtained with the Fear of Pain Questionnaire III [33] in individuals 18 years of age or older and with the Fear of Pain Questionnaire, Child report, in individuals from 6 to 17 years old [34]. Participants will complete the cold pressor test to provide a behavioral indicator of pain sensitivity [35].

### Data analysis plan

Hypotheses 1a, 2a, and 3 will be examined using SPSS software, as detailed below. Mplus software [36] will be used to address the remaining hypotheses. Note that throughout the analyses, an interaction between the two between-subjects factors is not of primary interest [19]. Rather, we anticipate that a particular combination of the groups, or the focal group, (i.e., pre-exposure with spacing and in multiple contexts) will have more favorable responses than each of the other groups. Consequently, we treat these between-subjects factors as one factor with four groups. Note that representing the four cells of the between-subjects factors in this way yields the same fit as a full between-subjects factorial model and readily enables us to assess the comparisons between the focal and other groups.

Hypothesis 1a: Spaced pre-exposure sessions, compared to no spacing, will (a) increase engagement of the proposed mechanisms (expected relevance and attention). Hypothesis 2a: Multiple context pre-exposure, as compared to pre-exposure in a single context, will (a) lead to stronger engagement of the proposed mechanisms (expected relevance and attention).

For Hypotheses 1a and 2a, changes expected in the proposed mediators should first be detected in the pre-exposure phase. To test these two hypotheses during the pre-exposure phase, a two-factor repeated measures MANOVA with one-between (pre-exposure group) and one-within-subjects (trial) factor will be run separately for the expected relevance and attention variables. A significant group × trial interaction indicates that mean responses across trials is not the same per group. If the interaction is present, follow-up analyses will focus on examining the plots of the mean change for each group and testing the degree to which the mean change from first to last trial differs between groups. For the conditioning phase, we will conduct a three-way repeated measures MANOVA separately for the two proposed mechanisms. For these models, pre-exposure groups will serve as the between-subjects factor with stimulus and trial being the within-subject factors, and the model will assess the main effects of each variable, as well as all-two-way and three-way interactions. Follow-up analyses depend on the nature of effects that are present (e.g., type of interactions or main effects). We anticipate differences will be larger between pre-exposure groups—predominantly at the early trials for stimulus CS^+^ _P_ —than for the other stimulus conditions. Thus, follow-up analyses will concentrate on the degree to which means differ by pre-exposure group and stimulus type for each trial using a Bonferonni-adjusted alpha, as well as examining plots of the mean response by stimulus type and trial for each pre-exposure group.

Hypothesis 3: Greater pain sensitivity will be associated with increased engagement of the proposed mechanisms of expected relevance and attention and increased LI.

To test the Hypothesis 3, pain sensitivity scores will be entered in the above analyses as a moderating variable. This will be done by testing interactions between pre-exposure and pain sensitivity.

Hypothesis 1b: Spaced pre-exposure sessions, compared to no spacing, will enhance LI of conditioned fear acquisition, recall, and retention. Hypothesis 2b: Pre-exposure in multiple contexts, as compared to pre-exposure in a single context, will enhance LI of conditioned fear acquisition, recall, and retention.

To investigate Hypotheses 1b and 2b, we will conduct mediation analysis to estimate direct, indirect, and total effects associated with the pre-exposure groups on the distal outcomes of conditioned fear acquisition, recall, and retention. In parallel mediation models, the pre-exposure groups are hypothesized to impact the mediators (expected relevance and attention), which, in turn, are hypothesized to affect a given fear outcome. Note that these path models do not include within-subjects variables as the scores for the mediating variables will be obtained from the first trial of the conditioning phase, where stimulus group differences are expected to be greatest, and scores from a given distal outcome will be computed as the average of the scores across specific trials in the recall and then retention phase. If the pre-exposure variable is found to interact with stimulus type in Hypotheses 1a or 2a, then this model will be estimated separately for each stimulus type to assess if mediation is present for each stimulus.

Parameters for the mediation analyses will be estimated in Mplus with maximum likelihood estimation and percentile bootstrapping (with 20,000 bootstrap samples), with the latter used to test for the presence of indirect effects. Bootstrapping is a recommended method for mediation analysis given that indirect effects are non-normally distributed [37–39], as each indirect effect is computed as a *product* of parameter estimates.

## Discussion

LI refers to when stimulus pre-exposure disrupts subsequent associative conditioning and thereby impairs learning. One domain in which LI has been observed is dental fear. Naturalistic studies find that undergoing non-aversive dental experiences prior to painful or traumatic dental experience(s) decreases the probability of developing significant and enduring dental anxiety from associative conditioning [13, 14]. Based on these findings, it may be possible to leverage LI to reduce the development of dental phobia and improve oral health [1]. To do so successfully, it is vital to clarify the mechanisms responsible for the LI of dental fear.

The proposed research will employee a new fear conditioning paradigm that uses a dental startle UCS for examining associative conditioning and LI [23]. The goal of the experiment is to modulate elements of pre-exposure to determine if these alterations potentiate two candidate mediators of LI. Specifically, based on a model of LI proposed by Hall and Rodríguez [18], we anticipate that spacing pre-exposure into multiple sessions and having pre-exposure occur in multiple contexts will increase engagement of the proposed target mechanisms of expected relevance and attention. These changes should, in turn, strengthen the LI of dental fear.

Currently, data supporting the LI of dental fear comes entirely from naturalistic self-report studies [13, 14]. As such, the proposed study will add to the scientific literature by providing experimental evidence regarding LI of dental fear as well as the mechanisms underlying this effect. To our knowledge, the study will be the first to experimentally assess the impact of pre-exposure session spacing and multiple context pre-exposure on the LI of dental fear. If the results are consistent with hypotheses, they would also show that the proposed mechanisms can be effectively modulated by researchers. Moreover, much of the evidence for the Hall and Rodríguez [18] model comes from non-human studies. The present research would expand the extant data on this model with a diverse human sample and with a novel dental startle paradigm.

There are several aspects to the proposed experiment that should be highlighted. First, it is expected that the research will include a diverse range of participants in terms of age (6 to 35) and minority group status (e.g., Black/African American and Hispanic/Latino individuals). This within sample variation should allow for a robust test of the hypotheses and do so with minorities who are most burdened by dental anxiety and oral health problems. Second, the study includes more objective indicators of one mediating variable (attention) and a behavioral indicator of fear learning (e.g., proximity of approach to the alien stimuli) which adds markedly to the other self-report measurements. Additionally, the inclusion of a fear retention test one week later is noteworthy, as many fear conditioning studies do not provide an extended fear assessment. This is a valuable addition that provides useful information for constructing interventions. Finally, the studies will also take a multi-method approach to assessing the role of pain sensitivity in the LI of dental fear.

The study is expected to provide data that will benefit future interventions. If pre-exposure prevention programs can be developed to reduce dental fear learning, it will be important to know whether such interventions will benefit from the inclusion of session spacing and context changes. This experiment, and others that identify strategies to strengthen LI, should be valuable in dampening the direct associative conditioning that causes dental phobia.

## Data Availability

No datasets were generated or analysed during the current study.

## Abbreviations

MANOVA: Multivariate analysis of variance
CS^−^: Unpaired control stimulus
CS^+^_NP_: Non-pre-exposed to-be conditioned stimulus
CS^+^_P_: Pre-exposed to-be conditioned stimulus
LI: Latent inhibition
ms: Millisecond
psi: Pounds per square inch
UCS: Unconditioned stimulus
